# Garlic ^(Allium sativum)^ Fresh Juice Induces Apoptosis in Human Oral Squamous Cell Carcinoma: The Involvement of Caspase-3, Bax and Bcl-2

**DOI:** 10.15171/joddd.2015.047

**Published:** 2015-12-30

**Authors:** Farrokh Farhadi, Salar Jahanpour, Kameliya Hazem, Amirala Aghbali, Behzad Baradran, Seyyed Mahdi Vahid Pakdel

**Affiliations:** ^1^Assistant Professor, Department of Oral and Maxillofacial Surgery, Faculty of Dentistry, Tabriz University of Medical Sciences, Tabriz, Iran; ^2^Dentist, Student Research Committee, Faculty of Dentistry, Tabriz University of Medical Sciences, Tabriz, Iran; ^3^Faculty of Medicine, Tabriz University of Medical Sciences, Tabriz, Iran; ^4^Associate Professor, Department of Oral and Maxillofacial Pathology, Faculty of Dentistry, Tabriz University of Medical Sciences, Tabriz, Iran; ^5^Associate Professor, Immunology Research Center, Tabriz University of Medical Sciences, Tabriz, Iran; ^6^Post-graduate Student, Department of Prosthodontics, Faculty of Dentistry, Tabriz University of Medical Sciences, Tabriz, Iran

**Keywords:** Garlic, apoptosis, squamous cell carcinoma

## Abstract

***Background and aims.*** There is no report on the apoptotic impact of Allium sativum L.(Garlic) on the oral squamous cell carcinoma (KB); hence, this study was designed to survey the apoptotic effects of garlic fresh juice (GFJ) on the KB cells.

***Materials and methods.*** MTTassay (MicrocultureTetrazolium Assay) was carried out to evaluate the cytotoxicity of GFJ on KB cells. Furthermore, TUNEL(Terminal deoxynucleotidyltransferase-mediated dUTP nick end labeling)and DNA fragmentation tests were performed to determine if GFJ is able to induce apoptosis in KB cells. Also a standard kit was used to assess caspase-3 activity in KB cells. Also western blotting was employed to evaluate the effect of GFJ on Bax:Bcl-2 ratio.

***Results.*** Significant cytotoxic effects were observed for the minimum used concentration (1μg/mL) as calculated to be 77.97±2.3% for 24 h and 818±3.1% for 36h of incubation (P < 0.001). Furthermore, TUNEL and DNA fragmentation tests corroborated the apoptosis inducing activity of GFJ. Consistently, after treating KB cells with GFJ(1μg/mL), caspase-3 activity and Bax:Bcl-2 ratio were raised by 7.3±0.6 and (P <0.001) folds, respectively.

***Conclusion.*** The results of this study advanced that GFJ induces apoptosis in the KB cells through increasing caspase-3 activity and Bax:Bcl2 ratio which could be attributed to its organo-sulfurcomponents.

## Introduction

 Nowadays, a significantly growing deal of attention has been focused on the use of natural compounds and dietary agents in order to prevent and treatment of a wide variety of medical complications such as cardiovascular disorders and cancer.^1,2^Life style, in case of cancer prevention, is a crucial factor. To put this into perspective, as a general concept, those who have had regular diets including enough amounts of fiber and antioxidants less likely will suffer from a specific type of cancer.^3^

 Among dietary agents the generally available ingredients which are widely being used by the majority of the world’s population with significant antioxidant an cancer preventive potentials are of utmost importance. For example, Allium sativum (Common name: garlic) from family Liliaceae is one of the most valuable edible plants.^4,5^The use of garlic as food, spice and traditional herbal medicinal backs to more than 4000 years ago.^6^

 There are a plethora of documents that have shed light on the cancer-preventing effect as well as other properties of garlic.^7^The pivotal ingredients responsible for many of the beneficial effects of garlic including its anticarcinogenic activity are known as organosulfur compounds.^8^

 According to meta-analyses of epidemiological studies garlic intake conversely correlates with the occurrence of some cancers. This conclusion was drawn that in individuals who consumed garlic regularly,gastric, stomach and colon cancers are significantly reduced in comparison to those who never or infrequently consumed it.^9^

 In a double-blind randomized clinical trial (RCT) in patients with colorectal adenomas a significant therapeutic effect was observed after 12 months consumption of aged garlic extract. In this study the size and the number of tumors in these patients noticeably reduced.^10^

 Moreover, after treating with the essential oil of *A. sativum*(0.11 μg/mL) complete inhibition was observed on the growth and mycotoxin biosynthesis of the fungus *Aspergillusversicolor*. In this study, the effects of garlic oil were attributed to its major compounds which were consisted of organosulfurs: diallyl-disulfide, diallyl-trisulfidemethylallyl-trisulfide, and methyl-allyl-disulfide.^11^

 A new organosulfur compound, garlicnin A, which was isolated from acetone extract of garlic was shown to suppress proliferation of tumor cellsthrough suppression of the tumor associated macrophages.^12^

 As there is no report on the effects of garlic on the squamous cell carcinoma, the current in vitro study was designed based on the evidence for the indisputable anticancer potential of *A. sativum* that prompted us to investigate the apoptotic effect of its fresh juice on the human oral squamous cell carcinoma cell line (KB).

## Materials and Methods

###  Plant Materials

 Fresh garlic was purchased from retail green grocery stores at Tabriz, Iran, and then was identified at the herbarium of pharmacognosy, Pharmacology Department, Tabriz University of Medical Sciences. On the day of experiments the bulbs of garlic were peeled and ground to obtain a fresh juice. Then the fresh juice was homogenized and was passed through 2 mm filters to be used for the experiments.

###  Total Sulfur Content of Garlic Juice

 To confirm the presence of sulfur containing compounds and to quantify the total sulfur content in the GFJ, a method previously described by Keisset al^13^was performed. The total sulfur content was measured in 1 mL of GFJ spectrophotometrically according to the abovementioned method. Calibration was done usingpotassium sulfate (K_2_SO_4_).

###  Cell Culture 

 KB cells (oral squamous cell carcinoma) were purchased from National Cell Bank (Pasteur Institute, Tehran, Iran). After Cell growthinRPMI- 1640 medium (Sigma, Germany) supplementedwith 10% FBS (fetal bovine serum) (Sigma, Germany),100 U/ml penicillin and 100 μg/ml streptomycin(Sigma, Germany). Incubation ofcells were performed in a humidified incubator containing 5% CO_2_ at 37ºC. At 80% confluence, cells were rinsed with phosphate buffered saline(PBS) 0.5% EDTA and harvested from 25 cm^2^ flasks using 0.25% trypsin/ EDTA solution (Gibco, UK). Then, the cells were sub cultured into 75cm^2^ flasks (Nunc, Denmark).

###  Terminal Deoxynucleotidyltransferase-mediated dUTPNick End Labeling (TUNEL)

 DNA fragmentation was detected TUNELtechniquewith the In Situ Cell Death Detection Kit, POD (Roche Diagnostics GmbH, Germany) according to the manufacturer’s instructions. Briefly, (1.5×10^5^) KB cells were sub-cultured into 6 well-plates and incubated for 24 h at 37°C and 5% CO_2_. The cells were treated with GFJ at concentrations for 50% inhibition of KB cells’growth (IC50) for 24h. Negative control cells were treated with the same final concentration of DMSO present in treated wells [0.2% (v/v)]. Having treated, 4% (w/v) paraformaldehyde in PBS (pH 7.4)was used for fixation of cells for 1 hour at room temperature and rinsed twice with PBS. Then, the fixed cells were incubated with blocking solution (3% H_2_O_2_ in methanol) for 10 min and rinsed with PBS. The cells were then incubated in permeabilization solution (0.1% Triton X-100 in 0.1% sodium citrate) for 2 min on ice. Subsequently, 50 μl of reaction mixture containing TdT enzyme and nucleotide was added to the cells and they were all incubated for 1 h at 37°C. After washingwith PBS, the slides were incubated with 50 μl converter-POD streptavidinHRP solution for 30 min, and rinsed three times with PBS. Finally, the cells incubated with DAB and cells were analyzed using light microscopy.

###  DNA Fragmentation Assay 

 Apoptosis has been definedbiochemically by the activation of a nuclear endonuclease that cleaves the DNA into multimers of 180-200 base pairs and can be visualized as an oligosomal ladder by standard agarose gel electrophoresis. KB cells were seeded in 6 wells plates and kept in CO_2_ incubator. KB cells were treated by GFJ in IC50 concentrations (5 μg/ml) for 24 h. At the end of incubation period, the cells were centrifuged for 1000 rpm for 3 minutes at 14°C. The pellet was re suspended in a lysis buffer (10 mMTris-HCI, pH 8.0, 10 mMNaCl, 20mg/ml Proteinase K, I0 mM EDTA,10% SDS), and incubated at 37°C. The pellet was dissolved in TE buffer (0.1 M Tris-HCl, pH 8.0, 10 mM EDTA). DNA samples were electrophoretically separated on 1.8 % agarose gel withethidium bromide (0.4μg/mL). DNA was visualized by a Ultra-Violet (302 nm) transilluminator. Untreated cells were used as control.

###  Evaluation of Caspase-3 Activity

 Caspase-3 activity kit (Beyotime Institute of Biotechnology, Haimen, China) was utilized for assessing the activity of caspase-3, as described by the manufacturer’s instruction. Briefly, KB cells were homogenized in 100 mL reaction buffer (1% NP-40, 20 mMTris-HCl (pH 7.5), 137 mMNad and 10% glycerol) containing 10 mL caspase-3 substrate (Ac-DEVD-pNA) (2 mM). After all treatments done(1, 5 and 10 μg/mL), lysates were incubated at 37°C for 2 h. The caspase-3 activities of the specimens were calculated using an ELISA reader at an absorbance of 405nm.

###  Western Blotting

 Protein expression levels were identified using western blot analysis. First,the separation of soluble proteins was done using lysis buffer (137 mMNaCl, 15 mM EGTA, 15 mM MgCl_2_, 0.1 mM sodium orthovanadate, 0.1%TritonX-100, 25 mM MOPS, 100 μMphenylmethylsulfonyl fluoride, and 20 μMleupeptin, pH=7.2). Subsequently, sodium dodecyl sulfate (SDS)-polyacrylamide gel electrophoresis was employed with a corresponding gel concentration using the discontinuous buffer system of Laemmli (Bio-Rad Laboratories, Richmond, CA). The resultant proteins were transferred to a polyvinylidene fluoride membrane(GE Healthcare, Amersham, Buckinghamshire, UK) and treated withimmunoblot analysis with antibodies to Bax and Bcl-2 (used at a 1/200 dilution, Abcam, Cambridge, MA, UK). The reaction was detected with enhanced chemiluminescence (GE Healthcare, Amersham, Buckinghamshire, UK). Afterwards, the membranes were washed and then reblotted with a β-actin antibody (1/2000, Abcam, Cambridge, MA, UK).ImageJ 1.62 software (National Institute of Health, Bethesda, Maryland, USA) was used for signal intensity measurement of each band.

###  Statistical Analysis 

 Descriptive data were reported as mean±SEM. One-way analysis of variance(ANOVA) was used to make comparisons between the groups with appropriate post-hoc tests (Student–Newman–Keuls). P<0.05 was considered as statistically significant.

## Results

###  MTT

**Figure 1.**
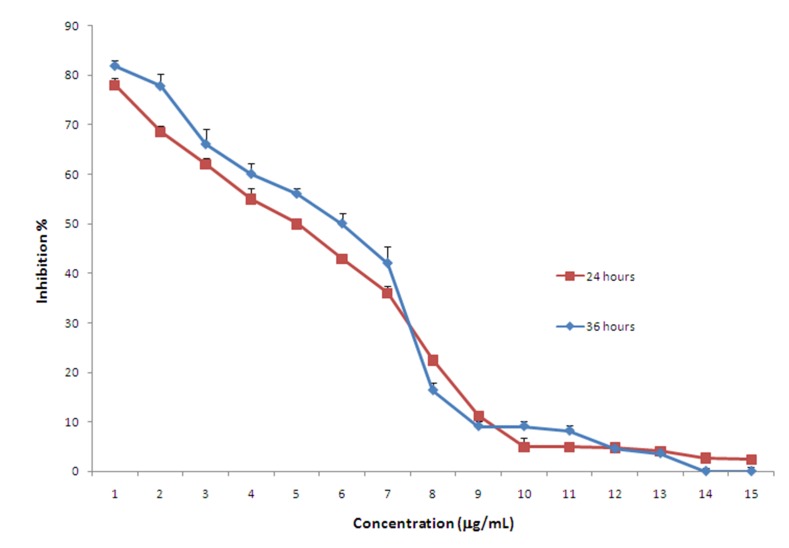
Inhibition percentages obtained after treating KB cells with garlic fresh juice at concentration range 1 to 15 μg/mL for 24 or 36 hours.

 Interestingly, following incubation of KB cells with 1 to 15 mg/mL of GFJ for 24 or 36 hours, it was revealed that GFJ was able to inhibit the proliferation of KB cells in a reverse dose-dependant manner. For both 24 and 36 hours of incubation, the maximum cytotoxic effect of GFJ was reportedwhen cells were treated with the minimum used dose of the juice (1μg/mL). The higher inhibition percentages calculated for the minimum used concentration (1mg/mL) were 77.97±2.3% for 24 h and 818±3.1% for 36 h of incubation (P <0.001). In addition, the IC50 values, the dose by which fifty percent of inhibition is obtained, were projected to be 5.8 and 4.9 and 5.8 mg/mL for 24 and 36 hours, respectively. Figure 1 depicts different concentrations of GFJ with which KB cells were treated and their respective inhibition percentages.

###  TUNEL 

**Figure 2.**
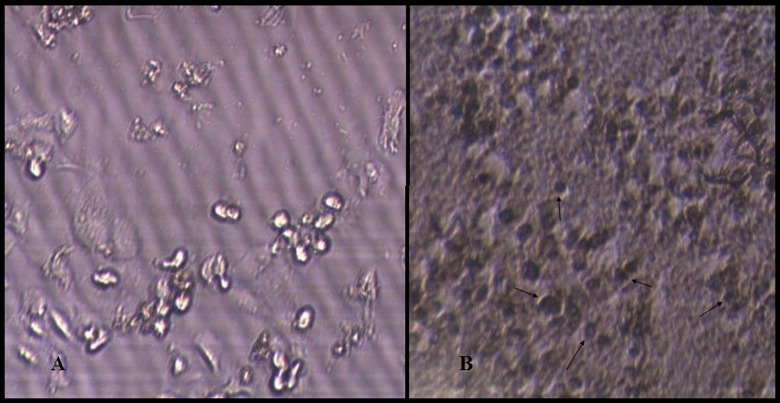
Effect of garlic fresh juice (GFJ) on the nuclei morphological changes detected by TUNEL test. A: control group (0.2% DMSO); B: treatment group (5μg/mL GFJ); arrows indicate cells showing signs of apoptosis (dark nuclei).

 After the treatment of KB cells with 5μg/mL of GFJ for 24 h, the apoptotic cells produced dark brown stained nuclei, whereas the non-apoptotic cells not stained as shownin the negative control cells treated with 0.2% (v/v) DMSO (Figure 2).

###  DNA Fragmentation

**Figure 3.**
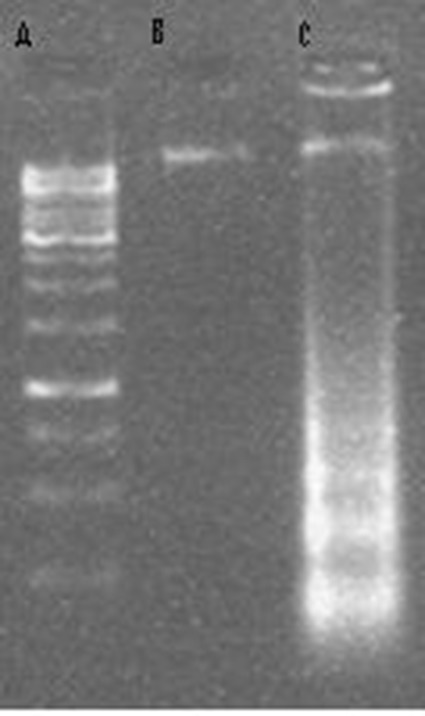
DNA laddering, DNA fragmentation performed on 1.8% agarose gel electrophoresis; A: 1000 bp marker; B: negative control (untreated cells); C: cells treated with GFJ (5μg/mL).

 As shown in agarose gel electrophoresis in Figure 3, increased DNA fragmentation was observed in KB cells after treatment with 5μg/mL (near to IC50) of GFJ.

###  Caspase-3 Activity

**Figure 4.**
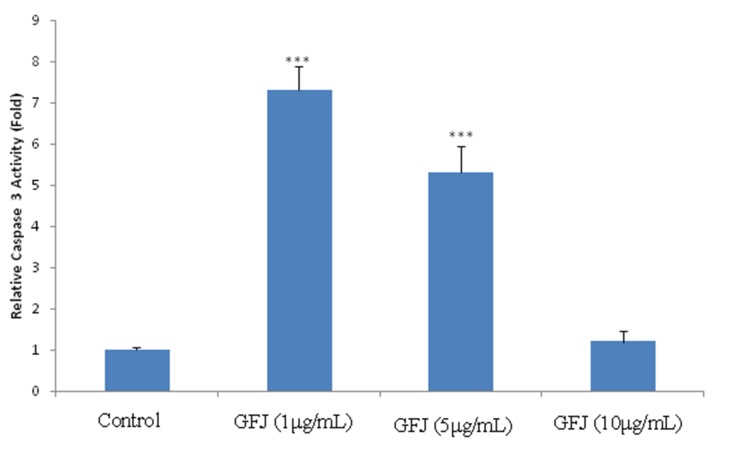
Effects of GFJ (garlic Fresh Juice) on the activity of caspase-3 in KBcells.Values are mean±SEM (n=3). ^***^ p <0.001 as compared with normal control group using one way ANOVA with Student- Newman-Keuls post hoc test.

 In comparison to untreated cells, after treating KB cells with 1, 5 and 10μg/mL of GFJ the activity of caspase-3 was increased by 7.3±0.6 (P <0.001), 5.3±0.65 (P <0.001) and 1.2±0.25 folds, respectively (Figure 4).

###  Western Blotting

**Figure 5.**
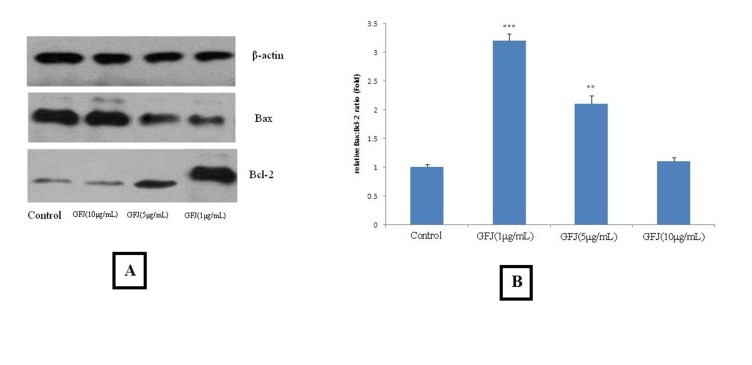
Effects of garlic fresh juice (GFJ)on Bax:Bcl-2 ratio in KB cells. KB cells treated with GFP (1, 5 and 10μg/mL) for 24 h. Then, cells were harvested and measured by Western blot analysis.A: Western blot analysis; B: Relative expression of Bax:Bcl-2 ratio calculated for each group. Compared with control group, a significant increase in Bax:Bcl-2 ratio was found in groups treated with 1 and 5 μg/mL GFJ in a reverse dose dependent manner.

 The results of western blotting were depicted in Figure 5. Accordingly, after treating KB cells with 1, 5 and 10 μg/mL, the Bax:Bcl-2 ratio was increased by 3.2±0.12 (P <0.001), 2.1±0.14 (P <0.01)and 1.1±0.07 fold, respectively.

## Discussion

 Based on the ample concrete evidence strongly suggesting anticancer effectsof garlic, this study was performed to determine the proapoptotic activity of garlic fresh juice (GFJ) on squamous cell carcinoma cell line (KB). Along with preliminary tests such as TUNEL and MTT to confirming the potential apoptosis inducing effect of GFJ in KB cells, more complicated assessments were done including caspase-3 activity assay and western blotting to project the Bax:Bcl-2 ratio. These assays were carried out to clarify the underlying mechanism by which GFJ enhances apoptosis in KB cells. Apoptosis or, inother words, programmed cell death is either a physiological or pathological event of crucial importance.^14^There is a great understanding of the molecular mechanisms as well as the proteins involved in this vital event. Of note, the balance between the proapoptotic protein Bax and the anti-apoptotic protein Bcl-2 is fundamental to apoptosis.^15^The ratio of these proteins is very determining; if the activity of Bax overweighs that of Bcl-2, it leads to the activation of its downstream effectors initiating or accelerating the process of apoptosis.^8^ One of the well-known downstream effectors of these proteins is a group of protease called caspases activation of which induces apoptosis.^16^

 In this study the presence of nucleosomal DNA fragments in GFJ-treated cells was revealed by TUNEL assay (Figure 2). This finding showed that the cytotoxic activity observed in the MTT assay was due to the apoptosis inducing property of GFJ.

 In the present work, the activity of caspase-3, as one of the main apoptosis inducers, was evaluated. Consistent with the results of MTT assay, GFJ at the minimum employed concentration (1μg/mL) was able to exert a greater effect on the activity of caspase-3 in comparison to other higher doses.

 Sulfur containing ingredients are of outmost importance. There is, throughout the literature, a tremendous body of evidence indicating a wide range of different properties for organosulfur compounds of garlic.^17^ For instance, it has been shownthat diallyltrisulfide (DATS), a sulfur containing ingredient of garlic, induces apoptosis in human breast cancer cells (MCF-7) via ROS-mediated activation of JNK and AP-1.^7^Moreover, when orallyadministeredat a concentration of 1–2 mg/day, thrice/week for 13 weeks, by DASTS markedly suppressed prostate carcinoma and pulmonary metastasis in mouse animal model without any side effects.^18^

 Additionally, DATS increased the H_2_O_2_ formation, lowered the thiol level and significantly suppressed cell proliferation and noticeably activated caspase 3 in human hepatoblastoma HepG2 cells.^16^Also it has been demonstrated that allicin, another organosulfur in garlic, induced apoptosis in murine T-lymphocytes (EL-4), as evidenced by formation of apoptotic bodies, nuclear condensation, DNA fragmentation, and by activating ofcaspases 3and 12,cytochrome C (cyt C), decreasing the mitochondrion membrane potential and increasing Bax:Bcl-2 ratio.^19^

 Moreover, S-allylcysteine, another sulfur containing ingredient of garlic, in human epithelial cancer cell line A2780, was demonstrated to inhibit migration and considerably lowered proteins involved in metastasis and growth including Wnt5a, p-AKT and c-Jun. Also S-allylcysteine decreased pro-caspase-3, Parp-1 and Bcl-2expression, and increased expression of active caspase-3 and Bax of which result in apoptosis induction.^20^

 Here, the total sulfur content of GFJ was projected to be 21.2 moles per mL. Taking into account many other studies signifying the clear and pivotal role of sulfur containing compounds of garlic, especially DATS, in inducing apoptosis through activation of caspases including caspase 3 and increasing the bax:Bcl-2 ratio, the apoptosis inducing effect of GFJ could be due to the presence of those compounds.^21^ However, the interesting finding in this study was the fact that all the effects were more significant when applying the lower doses of GFJ.This can be justified by considering many possibilities of which the probable antagonistic effects of some other compounds in the higher doses is of note. However, to clarify the certain causes of this exception further molecular studies are necessary.

 Taking into account the apoptosis promoting effect of garlic fresh juice, garlic would be acandidate of outmost value for treatment and prevention of the oral squamous cell carcinoma. However, it is suggested that further in vitro and in vivo investigations are carried outto identify the main ingredients of garlic juice which at low concentrations induce apoptosis and on the other hand, to find out why at higher concentrations this effect decreased.

## Conclusions

 The results of this study showed that GFJ induces apoptosis in the KB cells through increasing caspase-3 activity.

## Acknowledgments

 The present study was supported by a grant from the Research Vice Chancellor of Tabriz University of Medical Sciences, Tabriz, Iran. The authors declare that they have no competing interests.
